# Stroke 1-2-0-3-6: rapid identification and timely action for stroke in China

**DOI:** 10.3389/fneur.2025.1537895

**Published:** 2025-04-02

**Authors:** Chen Chen, Ziwei Hou, Li Li, Ruiying Wang, Hong Liu, Hui Shen

**Affiliations:** ^1^Shanxi Cardiovascular Hospital, Taiyuan, Shanxi, China; ^2^Shanxi Medical University, Taiyuan, China; ^3^Department of Neurology, Changzhi No. 2 People's Hospital of Shanxi Province, Changzhi, Shanxi, China; ^4^Department of Neurology, Shanxi Cardiovascular Hospital, Taiyuan, Shanxi, China

**Keywords:** stroke, 1-2-0-3-6, rapid identification, timely action, China

## Abstract

In 2016, doctors in China proposed the Chinese version of FAST, called Stroke 120, to improve stroke identification and response in the Chinese population. And we modified Stroke 120 by adding “3” and “6” which aimed to reduce the prehospital delays and improve the prognosis of patients with stroke.

Stroke is the leading cause of disability and death worldwide, and the incidence of stroke is higher in China than in most other countries (1). In 2007, American scholars proposed the Facial drooping, Arm weakness, Speech difficulties and Time to call emergency services (FAST) stroke educational tool (2). In 2016, doctors in China proposed the Chinese version of FAST, called Stroke 120, to improve stroke identification and response in the Chinese population (3).

The meaning of Stroke 120 is as follows: number 1 represents “check whether the face is asymmetrical;” 2 represents “examine the two arms for weakness”; and 0 represents “(absence of) clear speech.” The detection rate of stroke by family doctors has increased by 12.5% in China (4). However, some shortcomings remain in this approach. First, “FAS” in FAST emphasizes the symptoms, “T” emphasizes time, and the word FAST itself again emphasizes immediate action. Although Stroke 120 emphasizes the symptoms and emergency helpline number, it ignores the important window for timely response. Furthermore, it seems that there are still many people who do not understand the meaning of “one face” and “two arms.”

Therefore, we modified Stroke 120 by adding “3” and “6.” Numbers 1 and 2 represent 1/2, emphasizing “paralysis or numbness of half the body;” and 0 still means “(absence of) clear speech.” However, three emphasizes the window for intravenous thrombolysis for cerebral infarction, i.e., 3 h, and 6 denotes the window for arterial thrombectomy for cerebral infarction i.e., 6 h (Figure 1). Finally, Stroke 12036 represents recognizing the symptoms and dialing 120 in 3 and 6 h.

**Figure 1:**
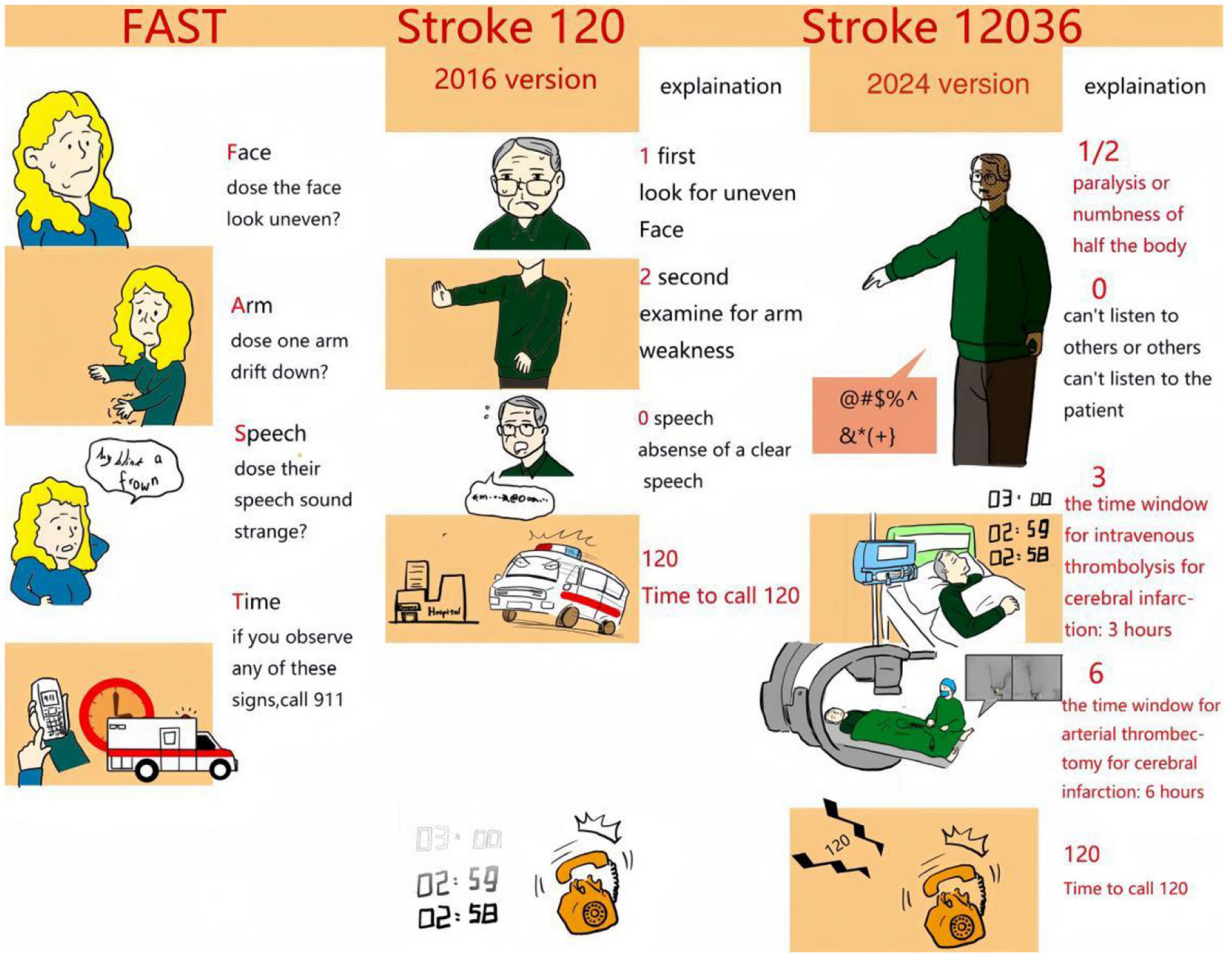
Stroke educational tool in USA and China. **Left panel**: FAST. **Middle panel**: Stroke 1-2-0. **Right panel**: Stroke 1-2-0-3-6. (In right panel, the differents from the previous version are shown in red).

Among these numbers, 120 is the emergency helpline number that Chinese people are familiar with; and 36 is a multiple of 12. Therefore, 12036 is easier to understand and remember than FAST. In addition to recognizing the symptoms by 120, there is a sense of urgency for 3 and 6 h. This tool was designed and released three times on Douyin account (the Chinese version of the video application TikTok). The video was played more than 400,000 times and shared nearly 20,000 times, indicating that the masses easily understood and accepted stroke 12036 (Figure 2).

**Figure 2:**
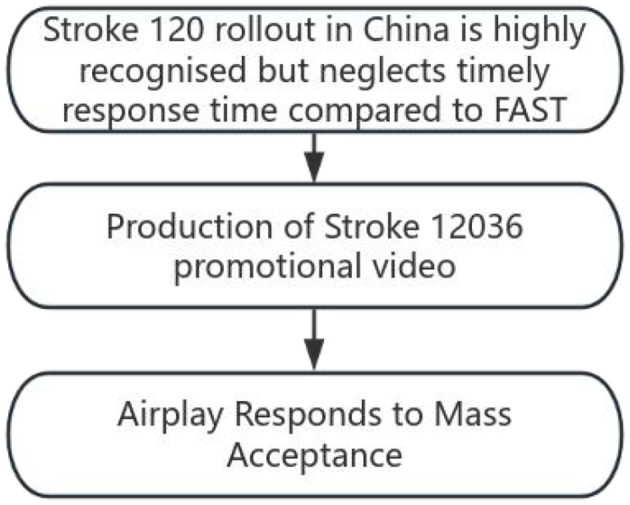
Study design flow chart.

We now plan to test this new strategy locally to establish a more suitable and final plan for China's family doctors and the general population. We aim to reduce the prehospital delays and improve the prognosis of patients with stroke (Figure 3).

**Figure 3:**
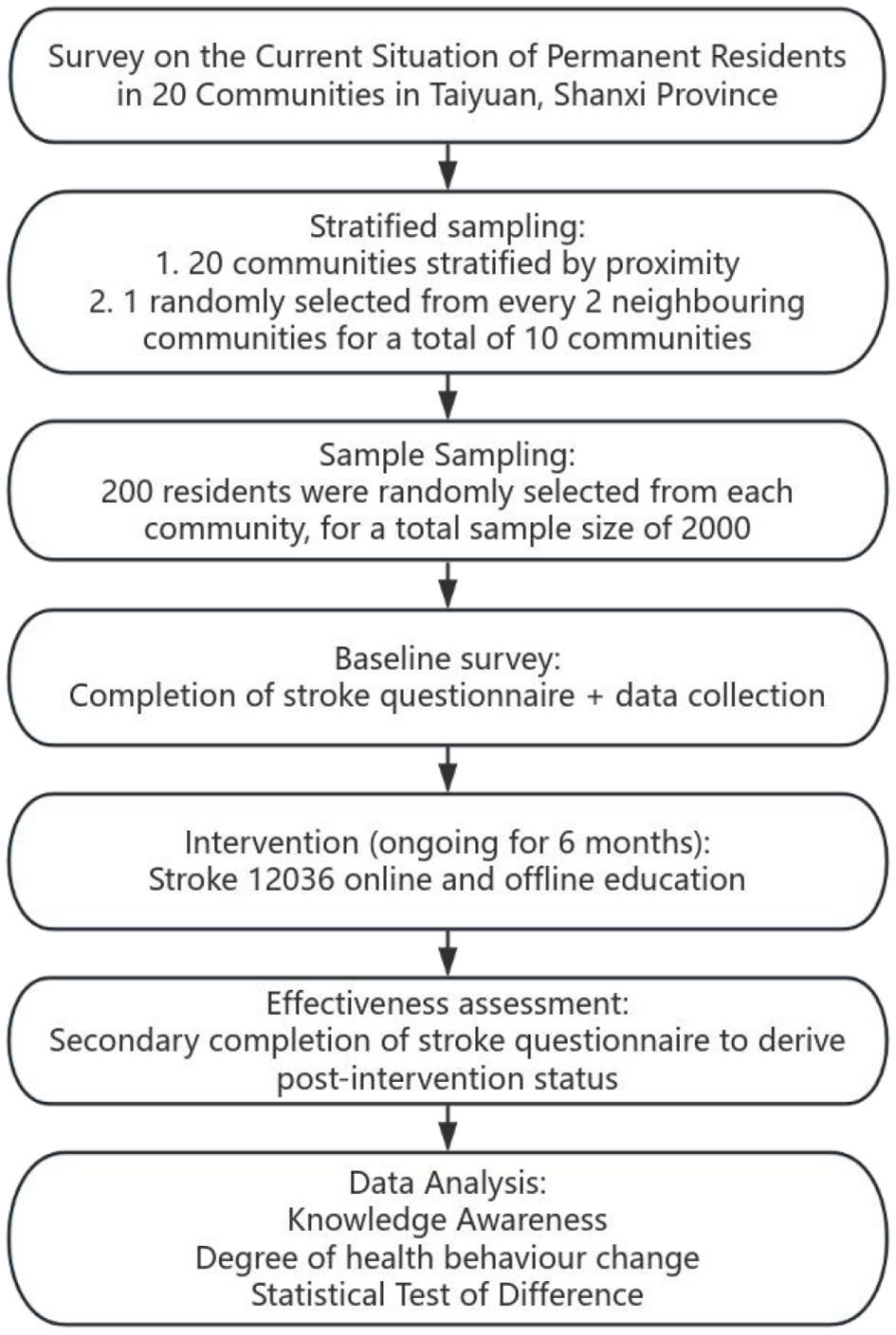
Flow chart of future research plans.

## References

[1] Nguyen TN Abdalkader M Fischer U Qiu Z Nagel S Chen HS. Endovascular management of acute stroke. Lancet. (2024) 404:1265–78. 10.1016/S0140-6736(24)01410-7 39341645

[2] Özdemir Z Acar E. YouTube as a source of recognizing acute stroke; progress in 2 years. BMC Public Health. (2024) 24:2208. 10.1186/s12889-024-19710-4 39138572 PMC11323591

[3] Zhao J Li X Liu X Xu Y Xu J Xu A. Changing the strategy and culture of stroke awareness education in China: implementing Stroke 1-2-0. Stroke Vasc Neurol. (2020) 5:374–80:374–80. 10.1136/svn-2019-000324 32350059 PMC7804060

[4] Liu X Weng Y Liu R Zhao J. Significant stroke knowledge deficiencies in community physician improved with stroke 120. J Stroke Cerebrovasc Dis. (2019) 28:104323. 10.1016/j.jstrokecerebrovasdis.2019.104323 31562040

